# Five years’ follow-up of dental fear and anxiety, experience of dental care and oral health behaviour in Swedish preterm and full-term adolescents

**DOI:** 10.1186/s12903-017-0431-0

**Published:** 2017-12-04

**Authors:** Susanne Brogårdh-Roth, Johanna Månsson, Karin Ridell, Lubna Alward, Kristina Hellén-Halme, EwaCarin Ekberg

**Affiliations:** 1 Department of Pediatric Dentistry, Faculty of Odontology, Malmö University, SE 205 06 Malmö, Sweden; 2 Department of Psychology, Lund University, Lund, Sweden; 3 Department of Oral and Maxillofacial Radiology, Faculty of Odontology, Malmö University, Malmö, Sweden; 4 Department of Stomatognathic Physiology, Faculty of Odontology, Malmö University, Malmö, Sweden

**Keywords:** Adolescent, Born preterm, Dental care, Oral health behaviour

## Abstract

**Background:**

There is rising concern about how preterm birth affects long-term health later in life. The various effects that preterm birth have on developmental outcomes, cognitive profiles and medical health may also affect levels of cooperation in the dental care situation in addition to general oral health and other oral health-related habits. Oral health is an integral part of one’s general health and well-being; however, less is known about how prematurity affects oral health and other related areas such as dental care, and including dental fear and anxiety (DFA) in individuals during adolescence and adulthood. This is considered of special interest to study, as preterm children during the preschool and school period were reported to have behavioural problems during dental treatments and less than favourable oral hygiene.

**Methods:**

A questionnaire was used of self-report design and structured into behavioural aspects relating to dental treatment, oral health-related factors, and medical health. This questionnaire at 17–19 years of age was a follow-up from 12 to 14 years of age and considered a predictor for planning future dental care for this group of patients. The 145 participating adolescents were all preterm, born between 23 and 32 weeks of gestation and 140 full-term controls, born ≥37 weeks of gestation.

**Results:**

Dental fear and anxiety, oral health behaviour, and intake of sweets and sugary drinks of 17–19-year old adolescents born preterm was comparable to that of the full-term control group. Medical health problems as well as the intake of sweets and sugary drinks increased from the time of early adolescence to late adolescence in both groups.

**Conclusions:**

Preterm as well as full-term adolescents between 17 and 19 years of age are satisfied with their dental care and display low prevalence of dental fear and anxiety (DFA). The findings in this study indicate that adolescents born very preterm and extremely preterm are well prepared for transition to dental care in adult life with expectations of being able to take responsibility for their oral health.

## Background

Despite recent advancements in medical care resulting in an increased survival rate, the risk remains that preterm children may develop a wide range of long-term complications in long-term health, cognitive function, and behavioural and emotional difficulties during childhood. The risk of impairment increases with decreasing gestational age, birth weight [1, 2] and neonatal complications [3]. Many of these have long-term consequences for health, growth, and development, with some problems continuing into adolescence and adulthood (e.g. behavioural difficulties, including long-term impairment in executive functioning and increased risk for psychiatric disorders [4–8]).

In the dental context, children and adolescents with a history of being preterm born constitute a new group of patients. Studies of behavioural problems in the dental situation expressed as uncooperative behaviour which lead to a delay in treatment, or even rendering treatment impossible, has shown that in stressful situations like dental examinations and treatments, behavior management problems (BMP) were more common in preterm children than in full-term controls during preschool and school period. Oral health problems, such as enamel defects of permanent teeth, less favourable oral hygiene, and gingival health were also more common in preterm children than full-term controls [9]. This leads to further concern about the long-term outcome of preterm children’s oral health, dental care and related factors, including dental fear and anxiety (DFA). In general, knowledge about young people’s views on their oral health during the transition to adulthood is limited [10]. Is prematurity a factor to highlight for dental health problems that individuals may face later in life? So far, studies have focused on children and young adolescents up to 14 years of age. To our knowledge, this study is the first to analyse oral health-related factors and dental care in older teenagers or young adults born preterm.

Adolescence is an age of transition to adulthood with expectations of being able to manage their own lives in different aspects, including taking responsibility for their general health and oral health. During this period, the adolescents evolve from being a receiver of care from parents to being a potential caregiver. A review of long-term outcomes of preterm birth concludes that preterm itself may be considered a chronic condition with risk for greater health needs in adult life [11]. Another study pointed out that a poor diet is a risk factor for cardiovascular disease, more specifically, that adults born preterm were reported to have a stronger preference for sweets than full-term born adults [12]. Such dietary habits may cause a risk for both medical and oral health problems in general. For adults born preterm, a gap of knowledge exists concerning the risk factors for poor oral health.

Given that adolescents are at an age when they are just about to leave the organized Swedish Dental Health Care system, the results from this survey may provide important information for future planning for dental care providers for this specific group of patients in transition to dental care in adult life.

The aims of the current study were to investigate experience of dental care, including DFA, satisfaction with dental care, oral health behaviour, dietary habits and medical health in preterm adolescents and full-term born controls at the age of 17–19 years in comparison with the same groups when they were between 12 and 14 years old [9].

Hypotheses:

- Due to preterm-born adolescents’ greater risk of developing behavioural difficulties, they have higher levels of DFA than full-term controls at follow-up at 17–19 years of age.
- Due to a well-functioning dental care system in Sweden, preterm-born adolescents are not considered special needs patients in their future dental care because, at 17–19 years of age, they are comparable to that of the full-term control group regarding oral health behaviour, dietary habits and satisfaction with dental care.

## Methods

### Study area

This study was conducted in southern Sweden, and preterm adolescents included were born at the University Hospitals of Malmö and Lund [9].

### Study population

All participants, both preterm and full-term originate from previous studies by Brogårdh-Roth 2010 [9]. In 2013 when the adolescents were 17–19 years of age, they were invited to participate in the current study. The original study sample included all adolescents born ≤32 weeks in the catchment area of Malmö-Lund from 1994 to 1996 (*n* = 192). With access to the Swedish Medical Birth Register, information on the children’s gestational age, birth weight, and number of siblings was collected from the Swedish National Board of Health and Welfare.

In the previous study, a control child born full-term was matched with every preterm child entering the study by age, sex, immigrant background (defined as at least one parent born outside the Nordic countries), dental clinic, and dental operator [13]. The same control children were invited to participate in the present survey.

In this study, the term preterm is used to describe children born at 32 weeks or earlier, very preterm to describe children born 29 to 32 weeks, and extremely preterm to describe children born 23 to 28 weeks.

Of the original 192 preterm adolescents, 18 declined participation for reason unknown, 21 were not reachable, and one was deceased. Further, seven preterm adolescents were unable to complete the questionnaire due to severe intellectual disabilities and were excluded. Consequently, 145 adolescents born preterm, of whom 113 very preterm and 32 extremely preterm, and 187 matched controls were included.

In the control group, 13 controls declined participation for reason unknown and 39 controls were not reachable, leaving 140 controls that agreed to participate in the present study. All the participating adolescents, except from 5 preterm and 3 controls, still lived in the same region as when aged 12–14.

#### Questionnaire

The questionnaire was of a self-report design and structured into the following parts. A similar questionnaire was used at the age of 12–14 years [9].

The head items in the questionnaire (Table 1) are as follows:

- Dental fear and anxiety (Children’s Fear Survey Schedule-Dental Subscale, CFSS-DS), [14]. Total scores range from 15 to 75, children/adolescents with a score ≥ 38 were categorized as having DFA according to Klingberg 1994 [15].
- Oral health behaviour was described in seven items [16, 17].
- Attitude to the dental care was described in two items: satisfaction with dental care, in general, and pain rating experienced during X-ray. The adolescent was asked to rate the pain intensity on a 100 mm Visual Analogue Scale. Rating ≥ 50 was classified as having pain in conjunction with radiographic investigations.
- Medical health included general health problems, disability, and daily medication. The following definition was used for chronic disease:
  - a disorder that is disabling, chronic or incurable, or
  - a disorder occurring at least three months during a one-year period and interfering with daily life functioning and/or needing treatment or special aids during at least three months, or
  - a disorder requiring hospitalization for at least one month or at least three periods during a one-year period [18].

**Table 1:**
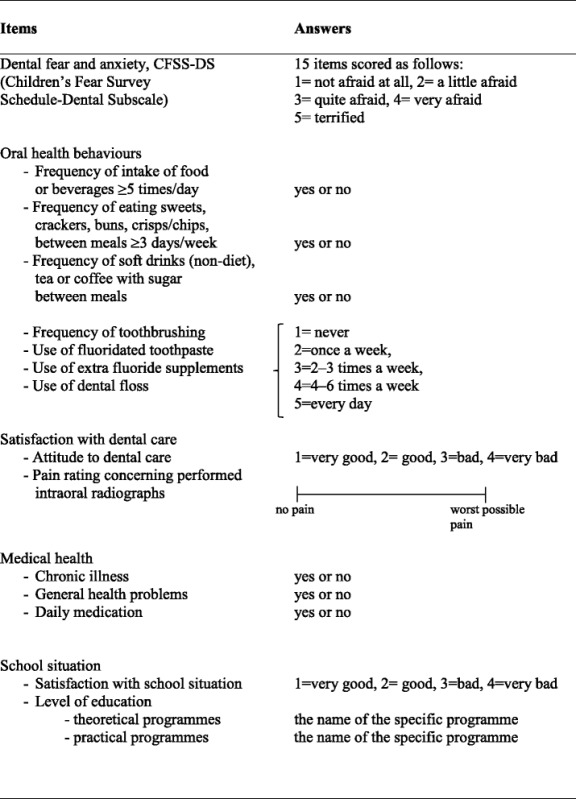
Items covered in follow-up questionnaire in preterm and control adolescents at 17–19 years of age

The definition of general health problems was having medical problems of lesser severity or duration, e.g., allergies or minor respiratory disorders.

- School situation consisted of questions regarding satisfaction with school situation and current education, classified as three-year theoretical or practical programs [19].

#### Analyses and statistics

The chi-square test was used for comparisons between the preterm and control adolescents, as well as for comparisons between sexes, between twins and singletons (within the preterm and control groups, respectively), and between the very preterm and extremely preterm groups. To assess the difference within the groups at 12–14 years and 17–19 years, Mc Nemar’s test was used. Mann Whitney test was used for comparisons between the groups for all respondents regarding CFSS-DS difference. The Statistical Package for the Social Sciences (SPSS) – version 16.0, 21.0 and 23.0 – was used.

## Results

The questionnaire was sent to 192 preterm adolescents and 192 controls. Of those, 145 preterm adolescents (75.5%) and 140 controls (72.9%) agreed to participate and returned the questionnaire.

Table 2 provides the characteristics of the study population. The mean age at the time of the questionnaire was for the preterm and control group 18.3 (range 16.8–19.8 years) and 18.4 (range 16.8–19.9 years) respectively.

**Table 2:** Baseline characteristics of very preterm adolescents (VPT), extremely preterm adolescents (EPT) and full-term adolescents at 17–19 years of age

	Preterm			
	Total	VPT	EPT	Full-term
	*n* = 145	*n* = 113	*n* = 32	*n* = 140
Sex				
Boys	67 (46.2%)	49 (43.4%)	18 (56.3%)	69 (49.3%)
Girls	78 (53.8%)	64 (56.6%)	14 (44.8%)	71 (50.7%)
Twins/triplets	47 (32.4%)	42 (37.2%)	5 (15.6%)	1 (0.7%)
Immigrant background				
Nordic	120 (82.8%)	98 (86.7%)	22 (68.8%)	115 (82.1%)
Immigrants	25 (17.2%)	15 (13.3%)	10 (31.3%)	25 (17.9%)
Mean gestational age in weeks	29.9	30.8	26.6	≥ 37
(range)	(24–32)	(29–32)	(24–28)	no data available
Mean birth -weight (gram)	1454	1604	922	3550^a^
range	615-2235	840-2235	615–1470	2100–4400

^a^ *N* = 119

Analysis of the dropouts shows a mean age for the preterm group of 18.1 (range 16.4–19.8), and for the controls 18.1 (range 16.4–19.8). Significantly more boys than girls among the preterm born adolescents declined participation. Among the controls there were no differences concerning sex.

### Questionnaire

#### Dental fear and anxiety (CFSS-DS score)

There were no significant differences regarding CFSS-DS scores between the adolescents in the preterm and control group at 17–19 years (Table 3). Those classified as having dental fear and anxiety (DFA - CFSS-DS score ≥ 38) were 5.5% in the preterm group and 2.1% of the controls. The subgroup analyses between the sexes within the preterm and control groups revealed slightly higher CFSS-DS scores for girls than boys in both groups at both 12–14 and 17–19 years. No other differences were revealed between very preterm and extremely preterm adolescents or between twins and singletons within the preterm group.

**Table 3:** *Dental fear scores* (CFSS-DS) and satisfaction with dental care in preterm (PT) and full-term adolescents (C) at 17–19 years and 12–14 years of age

	17–19 years of age			12–14 years of age		
	PT	C	*P*-value	PT	C	*P*-value
	*n* = 145	*n* = 140		*n* = 109	*n* = 108	
CFSS-DS						
mean	21.41	21.58	0.196^a^	21.89	21.57	0.820^a^
SD	7.68	6.82		6.37	5.97	
median	18.00	20.00		20.40	20.00	
range	15–48	15–61		15–47	15–45	
Satisfaction with the dental care	127 (87.6%)	117 (83.6%)	0.334^b^	105 (96.3%)	105 (97.2%)	0.710^b^
­ Very good	50 (34.5%)	56 (40.0%)		51 (46.8%)	58 (53.7%)	
­ Good	77 (53.1%)	61 (44.6%)		54 (49.5%)	47 (43.5%)	
­ Bad	18 (12.4%)	23 (16.4%)		4 (3.7%)	3 (2.8%)	
­ Very bad	0 (0%)	0 (0%)		0 (0%)	0 (0%)	

^a^MannWhitney test, ^b^Chi-square test

CFSS-DS items ranking at 17–19 years did not differ from 12 to 14 years. The items “Injection” and “Dentist drilling” were the most stressful items in both groups on both occasions. There were no statistically significant differences for any of the items between preterm and controls at either 12–14 or 17–19 years of age.

#### Satisfaction with dental care

In the preterm group, 127 (87.6%) graded their satisfaction with dental care as very good or good, compared with 117 (83.6%) in the control group. At 12–14 years of age, the corresponding figures were 96.3% in preterm group and 97.2% in control group (Table 3).

#### Oral health behaviour

There were no statistically significant differences in oral health behaviour between preterm and controls from 12 to 14 years to 17–19 years of age. Further, subgroup analyses revealed no statistically significant differences between the sexes within the two preterm groups, or between twins and singletons within the preterm group.

Twice as many both preterm and control adolescents at 17–19 years reported snacking (sweets, crackers, etc.) between meals ≥3 days/week compared with at 12–14 years (preterm, 43.4% vs. 18.3%; *p* = 0.001) (controls, 49.3% vs. 24.1%; *p* = 0.009) (Table 4). Similar figures were reported for drinking sugar containing drinks between meals (preterm, 48.9% vs. 29.4%; *p* = 0.011) (controls, 52.9% vs. 29.6%; *p* = 0.003) (Table 4).

**Table 4:** *Frequency of daily intakes, sweets and soft drinks* in preterm (PT) and full-term adolescents (C) at 17–19 years and 12–14 years of age. Chi-square test

	17–19 years of age			12–14 years of age		
	PT	C	*P*-value	PT	C	*P*-value
	*n* = 145	*n* = 140		*n* = 109	*n* = 108	
Eating or drinking ≥ 5 times/day	60 (41.4%)	60 (42.9%)	0.801	51 (46.8%)	46 (42.6%)	0.534
Eating candy, or similar snack foods between meals ≥3 days/week	63 (43.4%)	69 (49.3%)	0.323	20 (18.3%)	26 (24.1%)	0.302
Drinking non-diet soda, lemonade or other sugar containing drinks between meals	71 (48.9%)	74 (52.9%)	0.511	32 (29.4%)	32 (29.6%)	0.965

Boys, 17–19 years in both preterm and control group dominated in frequency for all dietary items. In the preterm group, there were significantly more boys than girls reported intake of food and drinks ≥5 times/day (52.2% vs. 31.6%; *p* = 0.014) and for drinking non-diet soda between meals (59.7% vs. 39.7%; *p* = 0.017). In the control group, significantly more boys reported snacking between meals ≥3 days/week (66.7% vs. 32.4%; *p* = 0.000).

#### Pain experienced during dental radiographs

Regarding pain during dental radiographic investigations, 49 preterm adolescents (33.8%) claimed experiencing pain (rating ≥ 50 on the VAS scale) compared with 52 controls (37.1%), a non-significant difference. Subgroup analyses revealed a significant difference between girls and boys in the preterm group (50.0% vs. 14.9%; *p* < 0.005) as well as in controls (52.1% vs. 21.7%; *p* = *p* < 0.005). Extremely preterm adolescents reported pain more frequently than in very preterm group, (40.6% vs. 31.9%) without a significant difference.

#### Medical health

Chronic illness had increased from 12 to 14 years and was significantly more reported by both preterm adolescents and full-term controls at 17–19 years (*p* = 0.035 vs. *p* = 0.039) (Table 5). A similar difference, though non-significant, was seen for daily medication (*p* = 0.057 vs. *p* = 0.581). Extremely preterm adolescents reported significantly more chronic illness than very preterm (34.4% vs. 17.7%; *p* = 0.042). No other statistically significant differences were seen for chronic illness, general health problems, or daily medication between the sexes within the two groups, or between twins and singletons within the preterm group.

**Table 5:** *Medical health* in preterm (PT) and full-term adolescents (C) at 17–19 years and 12–14 years of age. Chi-square test

	17–19 years of age			12–14 years of age		
	PT	C	*P*-value	PT	C	*P*-value
	*n* = 145	*n* = 140		*n* = 109	*n* = 108	
Chronic illness	31 (21.3%)	13 (9.3%)	0.005	11 (10.1%)	4 (3.7%)	0.064
­ neuropsychiatric disorders	10	6				
­ cerebral palsy, without intellectual developmental delay	9					
­ heart disease	3					
­ deafness	3					
­ diabetes	2					
­ severe asthma	2	2				
­ polycytotic syndrome	1					
­ malformed esophagus	1					
­ psoriasis		1				
­ alopecia		1				
­ vision loss		2				
­ epilepsy		1				
General health problems	45 (31.0%)	33 (23.6%)	0.158	34 (31.2%)	21 (19.4%)	0.047
­ allergies or minor respiratory disorders						
Daily medication	33 (22.8%)	17 (12.1%)	0.040	14 (12.8%)	7 (6.5%)	0.113

#### School situation

The majority of preterm and control adolescents reported satisfaction with their school situation at both 17–19 years (93.8% vs. 96.4%) and 12–14 years (97.2% vs 98.1%). With the former, 95.9% of the preterm group and 97.9% of the full-term control attended national programs at ordinary high school programs. Fewer in the preterm group followed a theoretical programme (67.6%) compared with full-term controls (75.8%); however, this is a non-significant difference.

## Discussion

### Main findings

In this survey, the majority of both preterm and controls rated their experience of dental care as very good or good, which is a sustaining result for the dental care system and may reflect a low overall rate of dental fear and anxiety (DFA). It appears that the transition from pediatric dentistry to adult dental care for all adolescents are well prepared. Results concerning DFA, oral health behaviour, and intake of sweets and sugary containing drinks of 17–19-year-old adolescents born preterm was comparable to that of the full-term control group. Thus, the proposed hypothesis that preterm adolescents would have higher levels of CFSS-DS scores than full-term control group is rejected. The expectation was that it would be higher in the preterm group because of the correlation to the reported increased risk for anxiety/depression during adolescence and young [6]. According to a meta-analysis, those born preterm or low birth weight were three times more likely to receive a diagnosis of a psychiatric condition, including anxiety and depression, in late childhood, adolescence and young adulthood [6]. Furthermore, different aspects of anxiety and depressive symptoms have been reported to be risk factors during early adulthood for dental fear and anxiety [20].

DFA refers to the patient’s experiences in dental care and is related to characteristics like temperamental factors and psychiatric problems as reported in preterm children [21, 22], and therefore, relevant to study. The prevalence of DFA is approximately 9% in a child population [23] and around 20% in an adult population [24]. In relation to this survey, the results showed 5.5% in the preterm group and 2.1% of the controls. In several population studies, CFSS-DS is the most frequently used measure of DFA, and the mean scores are lower than previously reported rates in adolescents [23] but fall in line with data from a study of 13–19-year-old Swedish adolescents, comparable as a reference group [25]. Also, the finding that girls had higher levels of CFSS-DS scores in both preterm and control groups is in line with previous population studies [23].

The hypotheses that adolescents born preterm are not to be considered as special needs patients in future dental care is partly rejected. Almost 50% of the adolescents reported consuming sweets and sugary drinks between meals several days per week, although in comparisons with full-term controls. The frequent consumption of soft drinks may increase the risk of caries and dental erosion [26], and it is linked to an unhealthy lifestyle in general. That more boys than girls reported a frequent intake of sweets and soft drinks several times per week is in line with a recent Swedish study of 16 year olds which named the consumption of sweets and soft drinks as a risk factor for being overweight and obese later in life, and therefore, a public health concern also from this perspective [27]. In comparison with early adolescence, the reported consumption of sweets and sugary drinks between meals several days per week in this survey had increased by about 100% in both groups. For the preterm group, with their increasingly compromized health status during adolescence, this reflects that preterm-born adolescents may be potentially at risk for oral health problems in adult life. Further, according to Sharafi et al. 2016 [12], less healthy dietary behaviours contribute to risk factors for cardiovascular disease in young adults born preterm.

The adolescent’s health status in this study increased twice from early adolescence (12–14 years) to late adolescence (17–19 years) in both the preterm and control group. These health problems included, for example, neuropsychiatric disorders, asthma, chronic lung disease, diabetes, and hearing impairments, with a prevalence of 34.4% in extremely preterm 17–19 year olds. These figures are in comparison with another Swedish study with extremely preterm 18-year-old adolescents reporting 37.6% chronic illness [28].

Dental procedures can be associated with pain, unpleasant feelings, and worry, and may result in stress and anxiety [29]. How the pain is perceived and rated relates to, for example, age, gender, and previous dental experiences [30]. Highly anxious children reported more pain than less anxious children [31]. Further, research suggests that preterm children’s experience of pain during neonatal intensive care might influence later pain sensitivity [32] and might contribute to adverse long-term physiologic and behavioural sequelae [33]. A radiographic examination is a common part of clinical dental examination and might be associated with more or less pain for everyone, even adults. The results from this survey showed that girls in both preterm and the control group rated significantly more pain than boys during radiographic exposure. This corresponds well with previous results reported by Krekmanova et al. 2009 [30]. To our knowledge, few studies have been carried out on pain sensitivity in the dental situation in preterm children. The finding that 41% of extremely adolescents and 32% of very preterm adolescents claimed pain associated with this examination suggests further exploration. The combination of common reported illness of differing severity and lower cognitive function, as frequently reported in preterm children, increases the importance of identifying pain management strategies [33], this may also include dental procedures.

### Study limitations

A limitation of the study is the small number of extremely preterm participants, making comparisons within the preterm group difficult; however, this falls in line with official statistics in Sweden. Further, the longitudinal study design made it impossible to include a larger number of extremely preterm individuals.

### Study strengths

This study was population-based and is a follow-up of a group surveyed from 3 to 14-years of age [9]. Consequently, the response rate of 76% in preterm and 73% in the control group is considered a satisfactory return. Another strength was the use of the same measurements and method of compiling data as in the previous study at 12–14 years. Further, the same examiner (SBR) carried out the studies on both occasions.

## Conclusions

The majority of the adolescents were satisfied with their dental care, and it seems like the transition from pediatric dentistry to adult dental care for all adolescents are well prepared. Although CFSS-DS, oral health behaviour, and intake of sweets and sugary drinks of 17-to-19-year old adolescents born preterm was comparable to that of the full-term control group, the number of chronic diseases and daily medication increased significantly in the preterm group. The combination of unhealthy dietary habits and chronic illness, as reported for preterm adolescents, needs to be further explored in preterm-born adults and may also be of interest from a medical point of view.

## Abbreviations

BMP: Behaviour management problems
CFSS-DS: Children’s fear survey schedule-dental subscale
DFA: Dental fear and anxiety
EPT: Extremely preterm born (gestational weeks 23–28)
PT: Preterm born (gestational weeks 23–32)
VPT: Very preterm born (gestational weeks 29–32)
